# Three Drug Combinations in the Treatment of Fit Elderly Multiple Myeloma Patients

**DOI:** 10.3390/jcm9113554

**Published:** 2020-11-04

**Authors:** Hélène Gardeney, Arthur Bobin, Cécile Gruchet, Florence Sabirou, Anthony Lévy, Laly Nsiala, Laura Cailly, Cécile Tomowiak, Jose Torregrosa, Niels Moya, Cyrille Hulin, Xavier Leleu, Stéphanie Guidez

**Affiliations:** 1 Service d’Hematologie et Therapie Cellulaire, and Inserm CIC U1402, 86000 Poitiers, France; helene.gardeney@gmail.com (H.G.); arthur.bobin@gmail.com (A.B.); cecile.gruchet@gmail.com (C.G.); sabirou.florence@hotmail.fr (F.S.); anthonyfoot90@hotmail.fr (A.L.); Lalynsiala@hotmail.fr (L.N.); Laura.cailly20@gmail.com (L.C.); Cecile.TOMOWIAK@chu-poitiers.fr (C.T.); Jose-Miguel.TORREGROSA-DIAZ@chu-poitiers.fr (J.T.); niels.moya@gmail.com (N.M.); Stephanie.GUIDEZ@chu-poitiers.fr (S.G.); 2 Service d’Hématologie et de Thérapie Cellulaire, CHU de Bordeaux, 33000 Bordeaux, France; Cyrille.hulin@chu-bordeaux.fr; 3 Service d’Hématologie et Thérapie Cellulaire, Universite de Medecine et Pharmacie, 33000 Poitiers, France

**Keywords:** elderly, multiple myeloma, treatment, frailty, survival

## Abstract

The multiple myeloma (MM) non transplant eligible (NTE) population is growing in line with the aging of the population in Western countries. Historically, this population has been known for having a greater risk of treatment related toxicity, and therefore drug development was slow and rather oriented towards the improvement of safety profile than the optimization of disease control. However, NTE MM patients, at least for the fit/non frail patients in recent years, seemed to have benefited more from a less palliative care to improve the depth of response and then prolong survival. NTE MM being a quite heterogeneous population, there are still a number of groups of patients that are in need of more efficient therapy, avoiding unnecessary toxicity, particularly for the frail patients. The use of triplet regimen with a melphalan-prednisone (MP) backbone has long been the standard of care for NTE MM, often dedicated to non-frail patients. New standards of care, triplet, and even quadruplet combinations, are emerging on the basis of the MP backbone but also on the more recently approved lenalidomide-dexamethasone (Rd) backbone. These developments were largely possible in line with the development of antibody-based immunotherapies (IT) in MM. The objective to improve outcomes with an acceptable safety profile will see other key therapeutic developments such as the dropping of dexamethasone early in the disease course or various attempts to allow permanent treatment discontinuation with a prolonged disease control. In that context, it is possible that immunomonitoring, minimal residual disease (MRD), and genomic risk-adaptation will become key elements of the treatment decisions on triplet-based regimens.

## 1. Introduction

Multiple myeloma (MM) typically develops among elderly patients with a median age at diagnosis of 70 years old, with an increased incidence with age. At the time of diagnosis 26% of MM patients are between 65 and 74 years old, increasing to 37% above 75 years old [1]. The prevalence of MM is expected to rise in the foreseeable future because of an aging population in Western countries and new therapeutic options have led to a longer survival of elderly MM patients [2]. Therefore, the percentage of elderly and very elderly patients with MM is expected to rise significantly. 

MM, a hematologic cancer characterized by malignant proliferation of plasma cells in the bone marrow is an incurable neoplasm, and as such the main goal for MM patients is to improve survival without a negative impact of treatments on the quality of life (QoL). Although the overall survival (OS) of elderly MM has improved with the advent of proteasome inhibitors (PI) and immunomodulatory drugs (IMIDs), advanced age / frailty remains a poor prognostic factor [3]. There is no evidence to support a different biology causing a worse prognostic in elderly MM. Indeed, looking at “high risk” MM (HRMM) defined by cytogenetic abnormalities, the incidence rate of t(4;14) is less frequent with aging (14% in ≤65, 10.9% between 66 and 74 and 8.3% in ≥75 years old), and the rate of del17p similar across age categories, around 6% [4].

The inferior outcome of elderly patients seems rather to be related to the frequency of frailty factors due to aging (comorbidities and polymedication, decreased physiological reserve, cognitive impairment) [5], all leading to an increased risk of treatment related toxicity. It is therefore impossible for most elderly newly diagnosed multiple myeloma (NDMM) patients to undergo an intensive treatment like triplet induction regimen followed by autologous stem cell transplant (ASCT), the current standard of care for younger MM transplant eligible patients. These patients are thus called non transplant eligible (NTE) rather than elderly MM.

Nevertheless, NTE is quite a diverse group of patients, with very heterogenous characteristics and various degrees of impaired function and numbers of coexisting comorbidities. Consequently, the biggest challenges in the care of NTE MM are to identify and characterize the degree of frailty, in order to determine the optimal treatment approach, a balance between safety profile and disease control. Further drug development is needed to ultimately reach an optimal safety/efficacity balance allowing no dose reduction across NTE MM patients whatever their frailty score. The development of immunotherapy has led to this path.

## 2. Frailty Scores for NTE MM

The assessment of the patients’ fitness or frailty associated with advanced aging is key to the treatment selection for NTE MM. Historically, treatment selection was based upon chronological age with the traditional cut-off of 65 years old and performance status quoted with the Karnofsky or ECOG indexes for instance. However, the multiplication of treatment options for NTE NDMM and the variety of frailty characteristics in that population led us to stratify this population in several subgroups taking into account the heterogeneity of their overall physical condition and their treatment objectives. Thus, in recent years, the frailty assessment has become a major challenge.

In this context, the concept of personalized medicine based on patients’ fitness or frailty profile emerged since the report of the European Myeloma Network in 2011 [1]. Besides clinical judgment, in order to help the physician to perform this careful geriatric evaluation, some scores specifically adapted to elderly MM have developed such as the International Myeloma Working Group Frailty Score (IMWG) [6] or the Revised Myeloma Comorbidity Index (R-MCI) [7].

The IMWG frailty score has first proposed a scoring system with a range from 0 to 5, based on age, comorbidities (quoted with Charlson comorbidity index) and the evaluation of dependence for the (instrumental) activities of daily living (ADL and IADL scores). The IMWG score has been validated in the FIRST trial [8] and fit patients had an awaited superior outcome compared to frail with a median progression free survival (PFS) of 24.0 versus 19.4 months (HR 1.36 95% CI 1.21–1.53, *p* < 0.0001) and a median OS of 70.1 versus 42.1 months (HR 1.86 95% CI 1.63–2.12, *p* < 0.0001), respectively.

On the other hand, the R-MCI takes into account age, the glomerular filtration rate (eGFR) estimated with MDRD formula, the presence of a pulmonary comorbidity and its severity, Karnofsky index, and cytogenetics.

Combining the 2 scores, it is possible to identify 3 prognostic groups in terms of risk of severe adverse events (AEs), risk of premature treatment withdrawal, progression free survival (PFS) and overall survival (OS): fit patients (IMWG score = 0, R-MCI ≤ 3), intermediate-fit (IMWG score = 1, R-MCI 4–6), or frail (IMWG score ≥ 2, R-MCI > 6). Other scores are under validation such as the UK Myeloma Research Alliance Risk Profile (MRP) [9] or the Hematology Oncology Frailty (HOF) scores [10].

The combination of the scores indicates if a patient is able to tolerate a full dose treatment or whether dose adaptation is preferable to limit treatment related toxicity. The 2014 consensus of the IMWG [11] has proposed upfront full dose conventional triplet based chemotherapy for fit NDMM aged between 70 and 75 years old and suggested reduced-dose therapy, regardless of their age, for patients older than 75 years old (or even 80 years old) or frail patients.

However, in the studies detailed below, patients are often only divided in frail/non frail groups so data on survival are rarely available for the intermediate fit group individualized by the frailty score mentioned above.

## 3. Objectives to Improve Survival in NTE MM

It has been shown that reaching the best response achievable, whenever possible a complete response (CR), was associated with improved survival, including for the eldest patients (≥75 years old) [12]. One step further, the ALCYONE trial [13] confirmed the value of deepening the response in the NTE population by showing that MRD negativity is associated with a diminution of the risk of progression or death, regardless of the treatment combination. These results call for a less palliative and a more active treatment approach for fit/non frail NTE MM to improve depth of response and prolong outcomes.

Transplant ineligibility has historically been above the cut-off of 65 years old, and more recently moved closer to 70 in real-world clinical practice [14,15,16]. However, non-frail/fit elderly MM patients, regardless of age, might be considered for upfront triplet-based induction and even possibly followed by ASCT, similarly to younger MM patients, while being cautious with drug management to avoid dose reductions that could impact the response. Indeed, there has never been any consensual age cut-off for transplant eligibility, since biological age and overall physical condition of elderly patients can differ from chronological age. ASCT has been evaluated in a retrospective fashion in the older MM population and it was concluded that ASCT can be safely performed in selected ≥65- and even >70-year-old MM patients. It has been shown that with adequate supportive care and control of complications, namely transfusions, antibiotics, and pain killers for mucositis, toxicity is similar with no excess in transplant-related mortality as compared to a younger cohort and with non-inferior outcomes (EFS, OS) or response [14,16,17,18].

The exact role of ASCT in the therapeutic arsenal in the care of elderly MM patients is not clearly defined, and only “selected” MM are considered transplant eligible beyond 65, and even more so beyond 70 years of age in real life. That clearly speaks to the need to develop better therapeutic options for NTE MM, including for non-frail/fit patients. Hope lies in the recent development of triplet-based combinations of proteasome inhibitors (PI) or anti CD38 immunotherapy (IT) plus IMIDs with induction and prolonged consolidation/maintenance to achieve a deep response, ultimately MRD negativity, hopefully associated to better outcomes.

## 4. Triplet-Based Combinations in NTE NDMM

All the data of trials in the NTE NDMM setting is summarized in Table 1.

### 4.1. Historical Melphalan Prednisone-Based Strategy

**MPT** (melphalan-prednisone-thalidomide) [19] has historically been the first triplet regimen to be approved for NDMM NTE in first line. However, the development of **VMP** (bortezomib-melphalan-prednisone, phase III VISTA study (VMP versus MP) [20]) has slowly replaced MPT. The addition of twice weekly IV bortezomib to MP has improved OS by 13 months and increased CR to 30% versus 4% with MP alone. The development of the sub-cutaneous (SC) bortezomib version has immediately improved the safety profile, particularly the neurotoxicity, but bortezomib can still not be given for a very prolonged time, although the continuous approach of most treatments has proved to improve survival.

Several attempts sought to improve the PI+ MP-based triplet combinations, starting with the CLARION study (**KMP** versus VMP) [21], evaluating the addition of carfilzomib (K), a second generation proteasome inhibitor of epoxyketone type in NTE NDMM. KMP failed to show a gain in PFS, the median PFS at 22.4 and 22.1 months respectively, *p* = 0.159. Several adjustments were proposed to improve the KMP regimen, including a greater carfilzomib dose concentration at either 20/56 or 20/70 mg/m^2^ as published in the Relapsed/Refractory MM setting (RRMM) [22,23,24], but to date only VMP remains the approved standard of care for the NTE MM.

### 4.2. Lenalidomide-Dexamethasone (Rd), a More Convenient Backbone for Triplet Combinations and Continuous Approach

The final analysis of the FIRST trial [25] confirmed the superior outcome with doublet oral therapy Rd until progression over triplet oral therapy MPT with a median OS of 60 months and a median PFS of 25.5 months with a gain of 4.3 months over MPT. In addition, the safety profile of Rd was appealing with grade ≥ 3 AEs appreciably less frequent with continuous Rd than with alkylating agent based MPT (70% versus 78%) and lower rates of hematologic events and peripheral neuropathy (NP). Thus, Rd has become a standard of care since 2017, widely popular due to being entirely oral, and MPT is no longer a standard of care in NTE NDMM.

Unlike for MPT**,** there was no head to head randomized trial comparing Rd to VMP. A 2017 meta-analysis [26] compared Rd to different melphalan-based combinations (VMP, MPT, and MP) and was in favor of a superior activity of Rd doublet therapy until progression, with an improved OS and PFS over melphalan-containing triplet therapy. However, the Larocca et al. study [27] focusing on NTE NDMM with high-risk cytogenetics (e.g., t(4;14), t(14;16), and del17p), induction therapy including bortezomib, and thus VMP combination, has shown prolonged survival over Rd with a median OS of 62.4 versus 43.2 months, respectively (HR 0.68; 95% CI 0.47–0.96).

Thus, Rd and VMP remain the two standards of care for NTE NDMM but need to be improved. The former might become the new backbone for future triplet-based combinations gaining in convenience and ability to be given continuously but has demonstrated the lack of survival improvement for high-risk NTE NDMM, and the latter hardly can be given continuously. In that context, two options emerged: improving Rd on a triplet basis benefiting from all the convenience of this backbone or trying to further improve VMP towards a quadruplet-based regimen, but then the quadruplet agent must have a remarkable safety profile.

### 4.3. The Addition of a Proteasome Inhibitor (PI) to the Rd Backbone

The first Rd based triplet regimen proposed was **VRd** (bortezomib-lenalidomide-dexamethasone) based on the SWOG-S0777 trial (VRd versus Rd) [28]. It was a 2017 US phase III randomized, open label study that evaluated the VRd induction regimen administered in 21 days cycles with bortezomib twice-weekly intravenously (IV), lenalidomide 25 mg from day 1 to 14 plus 20 mg dexamethasone in NDMM without immediate ASCT planned, for 8 cycles followed by continuous Rd until progression. The median PFS was 43 months in the VRd group with a gain of 13 months compared to Rd (*p* = 0.0018) and the median OS was improved by 11 months. Among HRMM, the gain of PFS was not statistically significant even though the median PFS was 38 months with VRd versus 16 months for Rd alone. Noticeably, the median age of the study’s population was 63 years old, patients being characterized as NTE for different reasons compared to “rest of the world” definition, with a greater proportion ≥ 65 in the Rd group (48% versus 38%). Finally, the improved PFS for ≥ 65 years of age in the VRd group was not statistically significant. The safety profile of this VRd regimen scheme was considered significant with 82% of AEs grade ≥ 3 for the VRd group (versus 75% in the Rd group) and around one fifth of patients (16%) had to stop treatment due to toxicity; but the increased toxicity profile is mainly due to the intravenous administration of bortezomib.

This increased toxicity led to elaborate dose adjustment with the “VRd lite” [29]. VRd induction regimen was given on 35 day cycles with bortezomib weekly SC, lenalidomide at a reduced dose of 15 mg from day 1 to day 21, and dexamethasone 20 mg. The induction regimen included 9 cycles and then patients were given consolidation with bortezomib and lenalidomide for 6 cycles; no maintenance therapy was mandatory. In this phase II study for NTE NDMM, the median age was 73 years old (range: 65–91). The primary endpoint was the overall response rate (ORR) after cycle 4 VRd and was 86%, with 66% ≥ very good partial response (VGPR), the median PFS was 35.1 months and OS was not reached. This modified bortezomib and lenalidomide schedule did not seem to negatively balance VRd’s survival benefit over Rd, with excellent tolerability in a frailer population. Indeed, only 4% of patients discontinued treatment due to toxicity and 74% completed induction. With regard to neurotoxicity, NP of any grade occurred in 62% of patients but only 1 patient experienced a grade 3 NP.

Although VRd has been established as a new standard of care, improving Rd with an acceptable safety profile, it still does not appear to be the best solution when it comes to improving outcomes for patients with high-risk disease [28], including with the dose and schedule modification [30] brought by the VRd lite schema.

In order to improve the PI + Rd regimen, increase the QoL and allow more continuous approach, another triplet attempt was developed with the use of ixazomib, **IRd** (ixazomib, a boronate acid oral-based PI lenalidomide-dexamethasone). Kumar et al.’s phase I/II study [31] evaluated IRd with ixazomib given at a flat dose of 4 mg on days 1, 8, and 15 of 28 day cycles for 12 cycles followed by ixazomib alone maintenance. The ORR was 80% including 32% ≥ CR. The median PFS was 35.4 (95% CI 17.84–44.12) and in ≥65 years old, 21.4 (95% CI 12.9–46) months. Although 86% of patients experienced ≥ grade 3 AEs, dose reductions due to AEs primarily impacted R (45%) and d (38%), mainly due to fatigue, NP, and diarrhea. The dose intensity of ixazomib was preserved with 96.3% of the planned dose administered. NP of any grade was reported in 45% but with only 2 patients reporting grade 3. Treatment withdrawal due to treatment toxicity was reported in only 14% of patients. The twice-weekly 3mg ixazomib schedule studied in Richardson’s phase I/II study [32] in younger patients with a median age of 63.5 years old, and not specifically NTE, demonstrated similar results overall. Sadly, the phase III, randomized, double blind, multicenter study TOURMALINE-MM2 trial (NCT01850524) evaluating IRd versus Rd in 700 NTE NDMM, did not meet its primary endpoint and failed to show a statistically significant improvement in PFS (HR, 0.83; *p* = 0.073, Takeda’s recent update [33]).

In an effort to continue to improve the PI + Rd regimen, carfilzomib was also studied in association to Rd. The previous encouraging results and outcomes in RRMM based on the ASPIRE study [34], and in first line for NDMM eligible for ASCT based on the FORTE trial [35], positioned **KRd** (carfilzomib-lenalidomide-dexamethasone) as a serious option in the race to the best Rd-based triplet combination in NTE NDMM. However, the results of the ongoing phase III ENDURANCE trial [36] (E1A11, NCT01863550) comparing VRD versus KRd as induction therapy in standard risk NDMM with a median age of 65 years, appeared disappointing in that regard. Indeed, the median PFS was superimposable across the two arms with 34.4 and 34.6 months respectively (HR 1.04; 95% CI 0.8–1.3: *p* = 0.74), with no differences seen based on age, presence or absence of t(4;14), or disease stage. Three-year OS was also similar in both arms, with 84% in the VRd arm (95% CI 80–88) and 86% in the KRd arm (95% CI 82–89). Regarding the toxicity profile, only 42% of patients completed the induction protocol in the VRd arm with 299 patients over 527 who discontinued the treatment early. On the other hand, fewer patients stopped treatment in the KRd arm with 202 discontinuations but it is unknown if the difference is statistically significant. The main reasons for treatment discontinuation in the VRd arm are AEs and in first place peripheral neuropathy, which is well known to be related to the use of bortezomib and even more likely to happen knowing that bortezomib was administrated either via IV or SC. NP rates are higher with VRd with 8% compared to < 1% in KRd. However, the KRd arm experienced a significantly higher rate of cardio-pulmonary and renal toxicities (16% compared to 5% in the VRd arm) and overall grade 3–5 AEs were more frequent in the KRd arm with 44% versus 22% with VRd (*p* < 0.001). So, it is likely that the higher rate of early treatment discontinuation in the VRd arm is due to NP which has a great impact on quality of life and despite a lower rate of severe AEs compared to KRd.

As a conclusion, triplet-based Rd regimens have proved a great benefit to NTE NDMM patients, although the data were mainly available for fit/non frail patients. VRd will likely remain the main standard of care across the PI + Rd options.

## 5. The New Area of Immunotherapy in NTE NDMM

Monoclonal therapeutic antibodies (mAb) have been developed for years in MM, essentially based on antibody dependent cell mediated cytotoxicity (ADCC), antibody dependent cellular phagocitosis (ADPC), and specific target monoclonal antibodies (MOAs). They have always failed in MM, until the recent development of antiSLAMF7 and CD38 targeting. The first fully humanized mAb targeting CD38, daratumumab, has certainly been the most exciting and successful treatment development in MM in recent years.

### 5.1. Daratumumab in Quadruplet VMP-Based Regimen

The anti-CD38 mAb has originally been approved in NTE NDMM in first line based on the results of the phase III ALCYONE trial [13]. The adjunction of daratumumab to VMP for 9 cycles followed by daratumumab alone until progression resulted in a significant improvement in outcome with an estimated 18-month PFS rate of 71.6% versus 50.2% in the VMP group (*p* < 0.001). The ≥ CR rate was greater in daratumumab-VMP, 42.6% versus 24.4% (*p* < 0.001). The safety of daratumumab appeared acceptable in the NTE population with most frequent ≥ grade 3 AEs being hematological and was comparable in both groups. The rate of treatment discontinuation was lower with daratumumab-VMP (4.9% versus 9% in the control group) even if the infection rate was higher (23.1% versus 4.7%) as well as infusion-related reactions (27.7% with 5% of grade ≥ 3 of grade 4). These infusion-related reactions are usually easily manageable with appropriate pre-medication with steroids, antipyretics, and leukotrien antagonists and tend to decrease after the first administrations, explaining the low rate (1.4%) of patients that stopped daratumumab because of this event.

Given the lack of improvement brought with the use of VMP regimen in NTE NDMM patients whose standard of care status was fading slowly over the years, the remarkable results of the quadruplet Dara-VMP regimen clearly brings VMP back into the race. However, one would still hardly believe a quadruplet regimen could be administered to all NTE patients and particularly the frail ones.

### 5.2. Daratumumab-Lenalidomide-Dexamethasone (DRd)

The phase III open label MAIA study [37], compared **DRd** until progression in 28 days cycles to Rd. Daratumumab was administered at a dose of 16 mg/kg once weekly during cycles 1 and 2, then every 2 weeks during cycles 3 to 6, and every 4 weeks thereafter. The median age was 73 years old (range: 45–90) with 44% ≥75 years old. Another 15% had “high risk” cytogenetic (HRMM). After a median follow-up of 28 months, the estimated 30-months PFS rate was 70.6% versus 55.6% respectively, and median PFS was not reached for daratumumab, compared to 31.9 months for Rd. The risk of disease progression or death with daratumumab was reduced by 44% (HR 0.56; 95% CI 0.43–0.73). DRd deepened the depth of the response with three times more negative MRD at 10^−5^ (24.2% vs 7.3%, respectively). This PFS benefit was also observed for ≥75-year-old patients with a HR of 0.63 (CI 95% 0.44–0.92). In contrast, the PFS benefit was less present in HRMM (HR 0.83 compared to 0.49 in standard risk). The safety profile of DRd is similar to previous data from ALCYONE [13] with mainly neutropenia (50.0% and 35.3%, respectively) and respiratory infections (13.7% and 7.9%, respectively). The infusion related reactions occurred in 41% of patients with only 3% of grade ≥ 3. The DRd triplet Rd-based combination is about to become a new standard of care upfront in NTE NDMM patients, and for now shows the best data ever reported in this population, with the exception of the HRMM population.

On a side note, a subcutaneous daratumumab infusion version will soon be available, that will clearly improve the QoL of patients, based on the phase III COLUMBA study [38] (NCT03277105). This study showed in RRMM that SC daratumumab at a flat dose of 1800 mg was not inferior to IV with a comparable safety profile. Although this study was not specifically designed for NTE NDMM patients, SC infusion would surely provide these patients a major benefit, particularly concerning QoL.

### 5.3. Elotuzumab, anti SLAMF7 mAb

The ongoing ELOQUENT 1 (NCT01335399) study is evaluating the adjunction of elotuzumab to the Rd backbone in NTE NDMM but it is hardly expected that its results would match those of DRd.

## 6. Triplet-Based Regimens in RRMM

Although no phase III studies specifically designed to compare standards of care were run in RRMM NTE patients, NTE patients are often recruited into studies performed in the RRMM setting. We have therefore synthesized some of the main data provided recently on regimens that incorporated an immunotherapy into standards of care.

The choice of therapy at each relapse is guided by the response to prior lines of treatment and noticeably whether the patient is refractory to lenalidomide.

Then, if non refractory, lenalidomide-containing regimens can be proposed at relapse. Randomized trials have already demonstrated deeper responses and improved PFS with the addition of a PI to the lenalidomide and dexamethasone doublet (Rd) in RRMM such as carfilzomib + Rd (KRD) in ASPIRE phase III study [34] (median PFS of 26.3 versus 17.6 months; HR 0.66; 95% CI 0.55–0.78 and 32% of CR versus 9%) and ixazomib + Rd (IRD) until progression in TOURMALINE-MM1 trial [39] (median PFS of 20.6 vs. 14.7 months; HR 0.74, 95%; CI 0.59–0.94).

Next, antibody containing, lenalidomide-based triplets were tested. Daratumumab + Rd (DRD) has been first investigated in RRMM patients in the POLLUX trial [40]. It has shown an impressive MRD negativity rate at 10^−4^ and 10^–5^ of 22.4% and the median PFS was not reached with DRD versus 18.4 months with Rd alone, HR 0.37 (95% CI 0.27–0.52, *p* < 0.01).

Then, anti SLAMF7 mAb elotuzumab + Rd (EloRd) was also tested in the phase III ELOQUENT 2 trial [41] with notably 20% of ≥75-year-old patients included. Median PFS with EloRd was 19.4 months (HR 0.70, 95% CI 0.57–0.85) and the benefit was persistent for ≥65-year-old patients (HR 0.65, 95% CI 0.5–0.85). In addition, EloRd was well tolerated in a population with renal impairment [42], particularly useful in the comorbid elderly MM population.

Pomalidomide has shown to be a solid backbone for combinations at relapse for RRMM refractory to lenalidomide or if lenalidomide has been previously poorly tolerated.

Similarly to Rd, a randomized trial has already demonstrated deeper response and improved PFS with the addition of a PI to the pomalidomide and dexamethasone doublet (Pd) in RRMM. The bortezomib + Pd (VPD) triplet evaluated in phase III OPTIMISMM trial [43] has shown a median PFS of 11 versus 7 months (HR 0.61, 95% CI 0.49–0.77) and 12% of CR versus 3%. In addition, carfilzomib + Pd (KPD) was studied in a phase I dose escalation trial [44] and showed a median PFS of 7.2 months (95% CI 3–9) and 16% of VGPR with an acceptable tolerance profile.

Next, antibody containing pomalidomide-based triplets were tested with CD38 targeting. Therefore, daratumumab + Pd (DPd) has been evaluated in an open label non-randomized phase 1b EQUULEUS; MMY1001 study [45] and showed interesting MRD negativity rates of 29% at 10^–5^ and a PFS of 8.8 months (95% CI, 4.6–15.4).

Afterwards, the second generation anti CD38 mAb, isatuximab + Pd (Isa-Pom-Dex) was evaluated in the phase III open ICARIA-MM trial [46]. Median PFS with Isa-Pom-Dex was 11.53 months versus 6.47 months with Pd alone (HR 0.596, 95% CI 0.44–0.81, *p* = 0.001) and it is consistent in sub-groups of patients ≥ 75 years old or with renal impairment. MRD negativity at 10^−5^ is reached in 5.2% with isatuximab.

Finally, elotuzumab + Pd (EloPd) was studied in the phase II ELOQUENT 3 study [47]. Noticeably, 63% of patients were ≥65 and even 22% ≥ 75 years old. Median PFS with EloRd was 10.3 months versus 4.7 with Pd alone (HR 0.54, 95% CI 0.34–0.86; *p* = 0.008). The tolerability of elotuzumab in association with Pd was excellent as serious adverse events (SAEs) were similar in both groups (53% with EloPd versus 55% with Pd alone).

In the RRMM setting, it is also possible to omit IMID drugs and so the challenge is to improve a PI backbone whether being bortezomib-based (Vd) or carfilzomib-based (Kd). Accordingly, the adjunction of a monoclonal antibody to the PI-dexamethasone doublet has been widely studied. The phase III CASTOR trial [48] evaluating daratumumab + Vd (DVd) has shown an improved survival with a median PFS of 16.7 versus 7.1 months (HR 0.31, 95% CI 0.25–0.40) and deeper response with 29% of CR versus 10%. However, if patients were already refractory to lenalidomide, the results are slightly disappointing with a median PFS under 12 months. On the contrary, the adjunction of elotuzumab to the Vd backbone (EloVd) failed to reach statistical significance in the improvement of PFS (HR 0.72, 95% CI 0.49–1.06) in a phase II study [49].

The daratumumab + Kd (DKD) triplet in the CANDOR study [50] has shown an improved survival rate with a median PFS at 17 months which is not reached in the DKD group versus 15.8 months with Kd alone (HR 0.63, 95% CI 0.46–0.85). However, it enrolled younger and fitter patients with a median age of 64 years old. DKD has also been evaluated in the phase 1b EQUULEUS (MMY1001) trial [51] with a notable weekly 20/70 mg/m^2^ schedule for carfilzomib. PFS was also not reached with DKD and seemed well tolerated, allowing further weekly schedule of carfilzomib with anti CD38 mAb.

Isatuximab + Kd (IsaKd) triplet evaluated in the phase III IKEMA study [52] showed, at a median follow-up of 20.7 months, a median PFS that was not reached with IsaKd versus 19.2 months with Kd (HR 0.531, 99% CI 0.318–0.889, *p* = 0.0007) and MRD negativity at 10^−5^ is reached in 29.6% with IsaKd.

## 7. Continuous Treatment Until Progression

One important growing concept in the field of myeloma care has been the increasing use of continuous treatment until disease progression with fixed duration regimen. The objective is to prolong the response or to improve its depth in order to delay the relapse and eventually lengthen the PFS and OS. This also focuses on the need to tailor medication management to improve the tolerability of treatment and avoid the negative impact of the dose intensity over time. To determine and to tailor the optimal duration of therapy has become another crucial point in the care of NTE MM patients more likely to suffer from the continuous triplet-based regimen approach. The demonstration of the role of continuous treatment until progression was first supported by the results of the FIRST study [25]. The PFS benefit of continuous over fixed duration of Rd for 18 cycles was demonstrated with a gain of almost 5 months. However, it failed to show any advantage on OS and could be related to the long use of corticosteroids and related adverse events such as infections and metabolic complications. However, maintenance with lenalidomide alone as in the UK Myeloma XI phase III study [53] also failed to show a benefit in OS over observation in the NTE NDMM arm with 3 years OS of 66.8% versus 69.8% (HR 1.02, 95% CI 0.80–1.29, *p* = 0.88). On the other hand, continuous triplet therapy in the NTE population could appear perilous with some drugs.

Various approaches have been studied to look into this ‘how to manage continuous treatment in NTE’ question. The first option would be to propose single agent maintenance to patients following triplet therapy, and several attempts were run in NTE patients. For instance, Kumar et al. have also evaluated the efficacy and the feasibility to continue ixazomib as a single agent maintenance after 12 cycles of IRd as a first line induction therapy for NTE NDMM [31]. They demonstrated that one third (32%) deepened their response with maintenance and the rate ≥ CR rate increased from 28% at the start of maintenance to 44% as best response on maintenance. This improvement in depth of response during maintenance was translated into prolonged outcome. Indeed, the median PFS was 37.2 months in NTE patients receiving maintenance versus 29.4 months in NTE patients not receiving maintenance. These results were persistent in the ≥65-year-old population with a gain in PFS even greater with 37 versus 21.5 months, respectively. The long-term tolerability of ixazomib is excellent with no treatment discontinuation due to AEs during maintenance and only 12% dose reductions. So continuous ixazomib compared favorably on toxicity profile to continuous Rd (in the FIRST trial) with a longer median duration of therapy with ixazomib. This highlights once more the limitations with steroids tolerance in elderly patients overall and the adverse impact on survival. This data was validated in the double-blind phase III TOURMALINE-MM4 trial (NCT02312258) of randomized single agent ixazomib maintenance versus placebo after 6 to 12 months of mainly PI-based induction in 706 NTE NDMM. Median age was 73 with 38% ≥ 75 years old [54]. Ixazomib maintenance has shown a 34% reduction in risk of progression or death and so, a PFS gain of 8 months with a median PFS of 17.4 months with ixazomib versus 9.4 months with placebo (HR 0.66, CI 95% 0.54–0.80, *p* < 0.001) at a median follow-up of 21.1 months. AEs during maintenance were mainly grade 1 or 2 and similar to previous studies, confirming a rather well tolerated safety profile. Ixazomib appears an interesting oral PI maintenance for NTE NDMM following cycles of triplet-based induction regimens.

The benefit of immunotherapy (IT) as monotherapy in maintenance has also been described in NTE NDMM in the ALCYONE trial [13]. It showed that the daratumumab maintenance increased the benefits of PFS following the quadruplet MPV + daratumumab regimen as induction. Indeed, the estimated PFS at 12 months (at the end of VMP induction) was 87% with Dara-VMP versus 76% with VMP alone and 72% versus 50% at 18 months, respectively.

So, maintenance therapy with PI or IT alone, namely oral ixazomib and daratumumab respectively, following triplet and even quadritherapy induction regimen in NTE NDMM patients has shown benefits in response rate and translated into prolonged outcomes with a suitable tolerability. However, relapse while on these continuous therapies, e.g., refractory disease to the given therapy, indicates the impossibility of reuse of these drugs at relapse.

The second option would be to favor the continuous triplet-based regimen, but this option cannot be offered to all regimens. The MAIA trial [40] has shown the possibility to continue triplet therapy until progressive disease (PD), independently of age and frailty condition, and in that regard is one of the unique regimens capable of managing triplet-based regimen until progression available to date. The development of IT-based triplet therapy manageable until progression could lead to further improvements of outcomes for NTE NDMM with a good tolerability and possibly have a lead across standards of care for NTE NDMM including for frail patients.

## 8. Future Perspectives for NTE NDMM

### 8.1. Triplet Combinations are Already Challenged by Quadritherapies with the Adjunction of Immunotherapy (IT) to a Triplet Regimen

With the previously discussed daratumumab + VMP (in the ALCYONE trial [13]) or in ongoing phase III studies evaluating daratumumab + VRd (CEPHEUS trial, NCT03652064) or isatuximab + VRD (IMROZ trial, NCT03319667). Daratumumab + ixazomib Rd is also investigated in a phase II (NCT04009109). Finally, elotuzumab + KRD (NCT02969837) is evaluated for NDMM in both transplant and non-transplant eligible patients.

These quadruplets carry the hope to improve the outcomes of HR NTE NDMM. However, there are several limitations already identified. First, VMP and VRd do not seem as appealing compared to DRd as a control arm. Secondly, quadruplets might be dedicated to non-frail NTE NDMM only due to the increased risk of toxicity. Thirdly, quadruplets will hardly be given for a very prolonged time. Other approaches will have to be anticipated.

### 8.2. Improving Safety of Frail NTE NDMM 

The current trend of continuous therapy with Rd is also challenged due to the well-known toxicity related to the long-term use of dexamethasone in NTE MM [55]. A growing concept in the care of NTE MM is the early discontinuation of dexamethasone to favor compliance with other drugs, but the Rd regimen then becomes R monotherapy. Therefore, the search for a new continuous association with lenalidomide has led to the evaluation of an anti-CD38 mAb to lenalidomide association in the continuous setting; phase III IFM-2017-03 (NCT03993912) is evaluating SC daratumumab + lenalidomide (DR) versus Rd for NTE NDMM frail patients.

It is appealing to imagine an association of an anti-CD38 mAb and lenalidomide as a new maintenance standard after an induction therapy of a triple or quadruple association with PI, IMID, and IT in the care of fit NTE NDMM patients in order to spare the use of corticosteroids while lengthening the response or even improving its depth.

### 8.3. The Field of Immunotherapy is Expending in RRMM 

With chimeric antigen receptor T cell (CAR-T), bispecific T-cell engagers (BiTEs), and antibody-drug conjugate (ADC) with belantamab mafodotin [56]. Concerning trending CAR-Ts, data on efficacity and safety in the older population are sparse. However, in a phase I study using bb2121 [57], an anti-BCMA CAR-T cell, inclusion criteria went up to 75 years old. So at least non-frail NTE NDMM patients should benefit from it. These new treatment options aim to obtain the quickest deepest response achievable, hopefully including HR.

### 8.4. Treatment Adaptation Depending on the MRD Status is Currently under Evaluation

The underlying hope is, in case of MRD negativity, the possibility of treatment discontinuation to avoid long term toxicity. Deepening the response could put an end to prolonged therapy which has real negative impact on QoL of patients.

## 9. Conclusions

NTE MM, historically called elderly MM, is the most represented, and highly heterogeneous population in MM. Therefore, it deserves specific standards of care, adapted to the frailty or fitness profile of each individual, with an aim to maintain the balance between safety and disease control.

In this population, patients’ characteristics, e.g., frailty evaluation and MM characteristics with biological prognosis at diagnosis (ISS and R ISS scores, high risk cytogenetic features) are equally important. In recent years there have been several publications to draw attention to the management of frail NTE and their greater risk of drug related toxicity due to their comorbidities and therefore the need for more personalized therapy [58] and prolonged EFS, a more optimal survival indicator over PFS.

With the potential for increasing disease-free life expectancy, the aim of deepening the response to treatments and therefore prolonging outcomes should be the goal in the care of non-frail/fit NTE NDMM, similarly to younger patients [58]. The emergence of new drug families, particularly immunotherapy, with front runners such as the anti-CD38 mAbs, and soon to come the CAR T cells, TCE/BITEs, etc., the objective of MM being a chronic disease and for some patients with “cure” is close.

The high-risk MM will remain the greater challenge to face despite all this progress, and the treatment paradigm shift to see a significant improvement to the outcome of these patients has yet to be discovered.

Although NTE NDMM including frail patients is the group of patients with the worse outcomes and with least progress, knowing the improvements in treatment exposed above, their outcomes might soon significantly improve and even become as good as for younger patients.

## Figures and Tables

**Table 1:** Results of trials upfront in non transplant eligible newly diagnosed multiple myeloma (NTE NDMM).

Studies	Regimen	RC (%)	MRD^−5^ (%)	PFS (Median, Months)	OS (Median, Months)	SAEs (%)	Discont (%)	High-Risk HR (95% IC)
VISTA	VMP ^(1)^	30	/	24	56.4	91	15	/
CLARION	KMP ^(2)^	25.9	/	22.3				/
FIRST	Rd ^(3)^	22	/	26	59.1	/	/	1.27 (0.81–2.01)
SWOG-S0777	VRD-Rd ^(4)^	15.7	/	43	75	/	/	/
Rd Lite	VRD-VR ^(5)^	32	/	35.1	nr	/	4	/
IRd Kumar	IRD-I ^(6)^	32	/	35.4	nr	52	14	/
ALCYONE	DaraVMP-D ^(7)^	42.6	22.3	nr	nr	41.6	4.9	0.78 (0.43–1.43)
MAIA	DRd ^(8)^	47.6	24.2	nr	nr	62.9	7.1	0.85 (0.44–1.65)

^(1)^ VMP = Bortezomib, melphalan, prednisone. ^(2)^ KMP = Carfilzomib, melphalan, prednisone. ^(3)^ Rd = Lenalidomide, dexamethasone until progression. ^(4)^ VRD -Rd = Bortezomib, lenalidomide, dexamethasone triplet induction followed by maintenance by lenalidomide-dexamethasone until progression. ^(5)^ VRD -Rd = Bortezomib, lenalidomide, dexamethasone triplet induction followed by bortezomib-lenalidomide. ^(6)^ IRD -I = Ixazomib-lenalidomide-dexamethasone triplet induction followed by maintenance by ixazomib alone until progression. ^(7)^ DaraVMP-DaraD = Daratumumab- bortezomib-melphalan-prednisone quadruplet induction followed by maintenance by daratumumab-dexamethasone until progression. ^(8)^ DRd = Daratumumab-lenalidomide-dexamethasone until progression. CR = complete response. MRD-5 = MRD negativity at 10^−5^. PFS = progression free survival. OS = overall survival. SAEs = serious adverse events. HR = high risk. 95%CI: confidence interval with an alpha risk of 5%. Discont. = discontinuation. Nr = not reached. Regimen = induction-maintenance.
